# Multi-omics analyses of cognitive traits and psychiatric disorders highlight brain-dependent mechanisms

**DOI:** 10.1093/hmg/ddab016

**Published:** 2021-01-22

**Authors:** Roxanna Korologou-Linden, Genevieve M Leyden, Caroline L Relton, Rebecca C Richmond, Tom G Richardson

**Affiliations:** MRC Integrative Epidemiology Unit at the University of Bristol, University of Bristol, Bristol BS8 2BN, UK; Population Health Sciences, Bristol Medical School, University of Bristol, Bristol BS8 2BN, UK; MRC Integrative Epidemiology Unit at the University of Bristol, University of Bristol, Bristol BS8 2BN, UK; Population Health Sciences, Bristol Medical School, University of Bristol, Bristol BS8 2BN, UK; Bristol Medical School, Translational Health Sciences, Dorothy Hodgkin Building, University of Bristol, Bristol BS1 3NY, UK; MRC Integrative Epidemiology Unit at the University of Bristol, University of Bristol, Bristol BS8 2BN, UK; Population Health Sciences, Bristol Medical School, University of Bristol, Bristol BS8 2BN, UK; MRC Integrative Epidemiology Unit at the University of Bristol, University of Bristol, Bristol BS8 2BN, UK; Population Health Sciences, Bristol Medical School, University of Bristol, Bristol BS8 2BN, UK; MRC Integrative Epidemiology Unit at the University of Bristol, University of Bristol, Bristol BS8 2BN, UK; Population Health Sciences, Bristol Medical School, University of Bristol, Bristol BS8 2BN, UK

## Abstract

Integrating findings from genome-wide association studies with molecular datasets can help develop insight into the underlying functional mechanisms responsible for trait-associated genetic variants. We have applied the principles of Mendelian randomization to investigate whether brain-derived gene expression (*n* = 1194) may be responsible for mediating the effect of genetic variants on eight cognitive and psychological outcomes (attention-deficit hyperactivity disorder, Alzheimer’s disease, bipolar disorder, depression, intelligence, insomnia, neuroticism and schizophrenia). Transcriptome-wide analyses identified 83 genes associated with at least one outcome (*P*_Bonferroni_ < 6.72 × 10^−6^), with multiple trait colocalization also implicating changes to brain-derived DNA methylation at nine of these loci. Comparing effects between outcomes identified the evidence of enrichment, which may reflect putative causal relationships, such as an inverse relationship between genetic liability towards schizophrenia risk and cognitive ability in later life. Repeating these analyses in whole blood (*n* = 31 684), we replicated 58.2% of brain-derived effects (based on *P* < 0.05). Finally, we undertook phenome-wide evaluations at associated loci to investigate pleiotropic effects with 700 complex traits. This highlighted pleiotropic loci such as *FURIN* [initially implicated in schizophrenia risk (*P* = 1.05 × 10^−7^)], which had evidence of an effect on 28 other outcomes, as well as genes which may have a more specific role in disease pathogenesis [e.g. *SLC12A5* which only provided evidence of an effect on depression (*P* = 7.13 × 10^−10^)]. Our results support the utility of whole blood as a valuable proxy for future studies analysing molecular datasets, but also suggest that conducting analyses in a tissue-specific manner may be more comprehensive.

## Introduction

Genome-wide association studies (GWAS) have discovered and replicated thousands of variants associated with cognitive and neurological traits and disease (1,2). However, findings from GWAS on their own do not provide insight into the biological processes that mediate the effect of these genetic variants. Developing our understanding of these molecular mechanisms is crucial in terms of disease prevention and treating disorders which affect normal brain function.

The majority of known trait-associated variants reside in the non-protein-coding regions of the human genome, with previous research implicating them in transcriptional regulatory mechanisms (3,4). This includes altering promoter and enhancer elements as well as enrichment in regions of the genome affecting gene regulation. With developments in microarray and sequencing technologies, data on genome-wide genotyping and gene expression from large samples are becoming increasingly available and have been used to identify genetic variants which robustly influence transcription (5,6). These variants are known as expression quantitative trait loci (eQTL) and have been shown to operate in a tissue-dependent manner (7). The GWAS variants responsible for cognitive and psychiatric trait variations are likely to exert their influence via gene regulation in the brain, given this organ’s role in the pathogenesis of these phenotypes (8). As such, using eQTL derived from brain tissue should be the most pertinent tissue type in terms of characterizing these effects. Furthermore, there are various cognitive traits and psychiatric disorders, which have been shown to be correlated either genetically or phenotypically [e.g. intelligence with ADHD (9) and Alzheimer’s disease (10,11) as well as neuroticism with schizophrenia, bipolar disorder and depression (12)]. As such, exploring whether these traits share a common eQTL derived from the brain tissue may highlight shared genetic architecture underlying such correlations.

We previously integrated tissue and cell-type specific eQTL data with findings from GWAS to investigate putative molecular mechanisms using the principles of Mendelian randomization (MR) (13,14). As with conventional risk factors, gene expression may be prone to confounding and reverse causation, which MR is robust to, by assessing whether a genetically predicted exposure has an effect on a complex trait or disease outcome. This approach is complemented by techniques in genetic colocalization to mitigate the likelihood that detected associations are driven by linkage disequilibrium between separate, but correlated, causal variants (i.e. one responsible for gene expression and the other for complex trait variation). As postulated previously, genetic colocalization at conditionally independent loci is necessary but not sufficient for causality (15).

In this study, we have applied a transcriptome-wide MR framework to discern whether the brain-derived gene expression may mediate the effect of genetic variants on eight different cognitive and psychiatric outcomes [attention-deficit hyperactivity disorder (ADHD), Alzheimer’s disease, bipolar disorder, depression, intelligence, insomnia, neuroticism and schizophrenia]. We subsequently applied the same analysis using eQTL data from a large collection of transcriptomes derived from whole blood (based on findings from the eQTLGen project). This was to elucidate brain tissue-dependent signals which would not have been detected using whole blood eQTL despite the large increase in sample size (brain eQTL, *n* = 1194, compared with whole blood eQTL, *n* = 31 684). Next, we integrated methylation quantitative trait loci (mQTL) data derived from brain tissue to discern whether there is evidence at the associated loci that DNA methylation may also reside along the causal pathway to cognitive or psychiatric outcomes along with the gene expression. Finally, to investigate pleiotropic effects, we have undertaken phenome-wide analyses by systematically applying MR to 700 different complex traits and disease.

## Results

### Uncovering transcriptome-wide signatures in brain tissue which putatively mediate genetic effects onto outcomes

We applied the principles of MR to identify genetically predicted effects between brain-derived gene expression and eight cognitive and psychological outcomes, using *cis*-acting single nucleotide polymorphisms (SNPs) (i.e. within 1 Mb distance of their associated gene) as instruments obtained from brain-derived eQTL datasets. There were 7443 genes whose expression could be instrumented by at least 1 *cis*-eQTL (*P* < 5 × 10^−8^) using the brain tissue. Analysing each of these genes with the eight cognitive and psychological outcomes identified 91 genetically predicted effects (across 83 genes with several genes linked with more than one outcome) which survived a *P* < 6.72 × 10^−6^ (i.e. 0.05/7443) as well as a genetic colocalization posterior probability of association (PPA) > 0.8 for single SNP analyses using the ‘coloc’ method (16) (Supplementary Material, Table S3). These included established loci in psychiatric genetics, such as *NEGR1* with depression (*P* = 2.15 × 10^−9^) as well as *RERE* and *FURIN* with schizophrenia (*P* = 4.43 × 10^−6^ and *P* = 1.05 × 10^−7^, respectively). There were also a host of loci which have not been linked as strongly to neuropsychiatric disorders in the literature. These included solute carrier family 12 member 5 (*SLC12A5*) (*P* = 7.13 × 10^−10^ with depression), TRIO binding protein (*TRIOBP*) (*P* = 3.59 × 10^−8^ with intelligence), *STX1B* and solute carrier family 25 member 12 (*SLC25A12*; both of which had a putative effect on neuroticism with *P* = 1.26 × 10^−8^ and *P* = 6.42 × 10^−6^, respectively). We include a table of full MR results (i.e. without thresholding for genetic colocalization) in the Supplementary Material, Table S4. We found that 38.7% of these results were supported by the evidence of colocalization. Performing a sensitivity analysis using an intelligence GWAS (i.e. based on fluid intelligence score) not adjusting for socioeconomic status (SES) produced similar results to those of Savage *et al*. (17) (Supplementary Material, Table S5). The results from our study were comparable to those detected by previous transcriptome-wide association studies. For example, similar to publications by Hall *et al*. (18) and Gusev *et al*. (3), we identified genes such as chloride channel 3 (*CLCN3*), *GATAD2A* and *ELAC2* but also identified novel genes not highlighted by these studies such as *NEURL*, *TRIM37* and *SDCCAG8* using MR and genetic colocalization.

**Figure 1:**
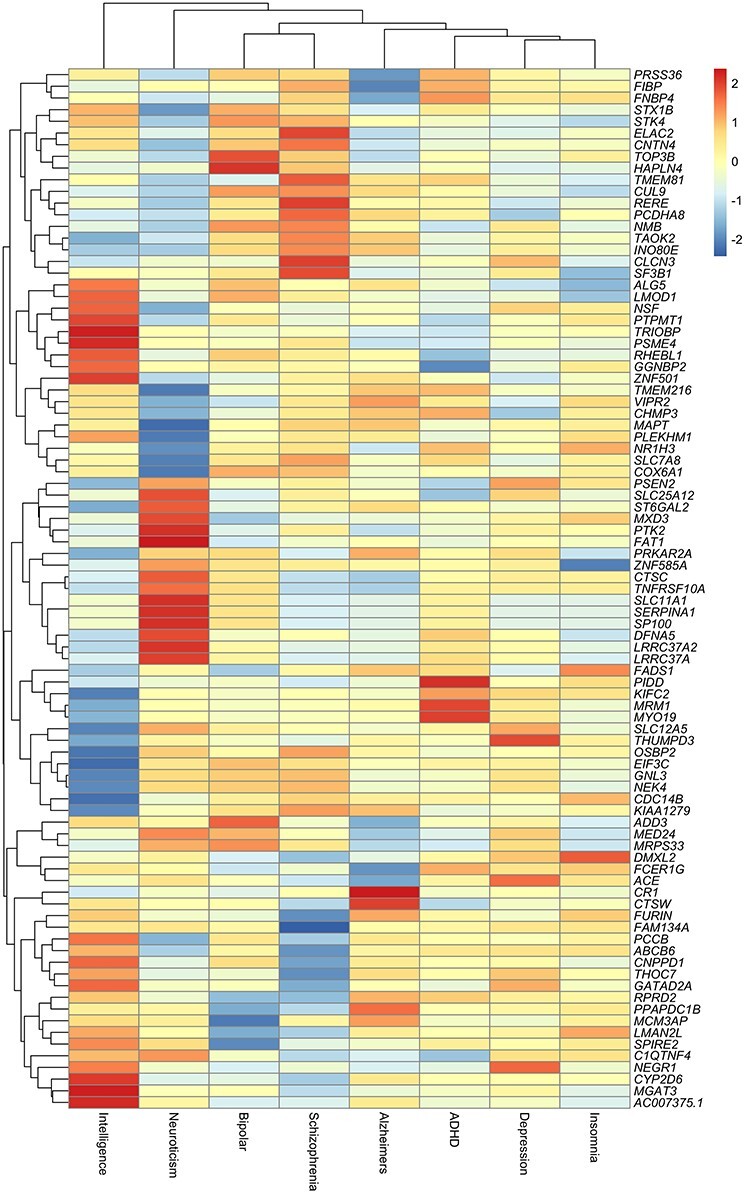
A heatmap illustrating MR effect estimates between the brain-derived gene expression for 83 genes and eight neurological and psychiatric traits and disease.

Subsequently, to assess the potential shared aetiology between the eight traits, we investigated how the genetically predicted effects of identified genes on all eight outcomes clustered based on Euclidean distance matrix computation (19). We did this by applying the R package ‘dist’ to compare the distances between our MR estimates for the same gene across the eight cognitive traits and psychiatric disorders. These results were therefore used to highlight the local genetic correlation which exists between the 83 loci identified in our analysis across each of the outcomes evaluated. A heatmap visualizing these results can be found in Figure 1.

This analysis highlighted clusters of genes whose shared effects amongst outcomes which may reflect causal relationships. For example, we observed evidence of enrichment for an inverse relationship between schizophrenia-associated genes and intelligence (Fig. 1), which may reflect the evidence postulating a causal relationship between schizophrenia genetic liability and lower cognitive ability in later life as presented by previous MR analyses (20). Likewise, there was enrichment of an inverse relationship between ADHD-associated genes and intelligence (Fig. 1), which has also been reported by a previous MR study (21). Therefore, these loci contain candidate genes where vertical pleiotropy may exist, such that their effect on schizophrenia liability through changes to gene expression may consequently have an influence on the measures of cognition.

**Figure 2:**
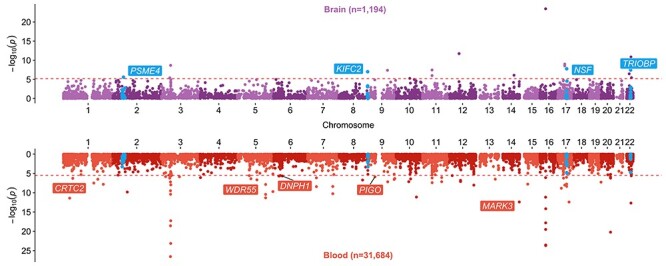
A bi-directional Manhattan plot depicting the transcriptome-wide effects for gene expression on intelligence. The top plot represents effects derived from brain tissue (purple, *n* = 1194), whereas the bottom plot illustrates the same analysis using whole blood data (red, *n* = 31 684). Genes surviving multiple testing (*P* < 6.72 × 10^−6^ in brain, *P* < 3.27 × 10^−6^ in blood) based on single SNP analyses were additionally subjected to genetic colocalization (PPA > 0.8). Loci highlighted in blue survived multiple testing in brain-derived tissue but not in whole blood (despite the large difference in sample size). Loci which are labelled in red on the inverse plot represent candidates that were not identified in our brain tissue analysis but may survive multiple testing corrections in larger samples.

Enrichment analyses provided evidence that the genetically predicted expression of the 83 identified genes were enriched for various regions of the brain compared with a background set of loci (Supplementary Material, Table S7). For example, the predicted expression of neuroticism-associated genes was enriched in the hippocampus (*P* = 8.61 × 10^−7^), amygdala (*P* = 3.61 × 10^−5^) and substantia nigra (*P* = 4.36 × 10^−5^). Genes associated with intelligence were enriched in the frontal cortex (*P* = 0.0001), cerebellum (*P* = 0.0005) and anterior cingulate cortex (*P* = 0.0004) (Supplementary Material, Fig. S3). There was also enrichment for the genes associated with bipolar disease in the hypothalamus (*P* = 0.0005), whereas the expression of insomnia-associated genes were overrepresented in the cerebellum (*P* = 0.0006).

### Discerning whether effects are brain tissue-dependent or identifiable using whole blood data

Applying our MR framework using whole blood eQTL data (*n* = 31 684) identified 260 genetically predicted effects (across 234 loci), which survived multiple testing corrections [*P* < 3.27 × 10^−6^ based on a maximum of 15 301 genes (Alzheimer’s disease) with at least one instrument in this tissue] (Supplementary Material, Table S8). Comparing effect estimates from our brain tissue analysis found that 53 of the 91 genetically predicted effects had a *P* < 0.05 in blood (Supplementary Material, Table S9). Therefore, 41.8% of these putative effects may have been overlooked by not using brain tissue based on this heuristic. This included several genes which appear to be predominantly expressed in brain tissue based on findings from the GTEx consortium, such as N-ethylmaleimide sensitive factor (*NSF*), protocadherin alpha 8 (*PCDHA8*) and *SLC12A5* (Supplementary Material, Figs S1–S3, respectively). Figure 2 provides an illustration of findings for intelligence as an outcome, where four loci [*PSME4*, kinesin family member C2 (*KIFC2*), *NSF* and *TRIOBP*] provided much stronger evidence of a genetically predicted effect using brain tissue than in whole blood despite the large difference in sample sizes (*n* = 1194 in brain tissue and *n* = 31 684 in whole blood). Conversely, 37 (14%; i.e. 37/260) genetically predicted effects from the whole blood analysis had an effect resulting in *P* < 0.05 in brain tissue, although these did not survive transcriptome-wide corrections (Supplementary Material, Table S10). However, this may be due to having lower power in the brain tissue-based analyses. As such, they may be worthwhile candidates for future studies to prioritize.

### Elucidating epigenetic mechanisms which may also play a role in trait variation and disease susceptibility

**Figure 3:**
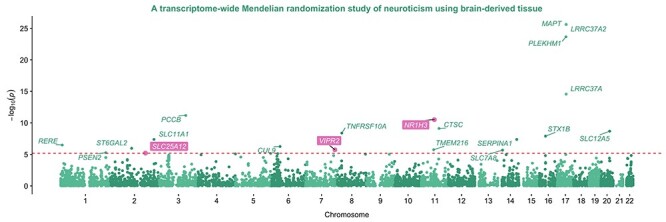
A Manhattan plot representing the transcriptome-wide associations between brain-derived expression and neuroticism. Genes surviving multiple testing (*P* < 3.27 × 10^−6^) based on single SNP analyses were additionally subjected to genetic colocalization (PPA > 0.8). Points highlighted in pink provided evidence from multiple trait colocalization analyses that brain-derived DNA methylation, as well as gene expression, share a causal variant at these loci with neuroticism susceptibility.

For effects identified in the initial analyses using brain-derived eQTL data, we conducted follow-up analyses to distinguish whether DNA methylation levels are also implicated in the risk of the examined traits/disease outcomes. We undertook multiple trait colocalization using the R package `moloc' (16) at each of the 83 loci identified in the brain tissue analysis to discern whether DNA methylation, gene expression and complex traits all shared a common causal variant. There was evidence that this was true at nine loci based on brain-derived DNA methylation data with a PPA > 0.8 (Supplementary Material, Table S11). Figure 3 depicts the results from the transcriptome-wide analysis of neuroticism, where highlighted loci provided evidence of a shared causal variant for DNA methylation, gene expression and neuroticism risk. These were *SLC25A12* (highest PPA = 0.96), *VIPR2* (highest PPA = 0.96) and *NR1H3* (highest PPA = 0.99).

### A phenome-wide evaluation of genes to highlight pleiotropic effects

**Figure 4:**
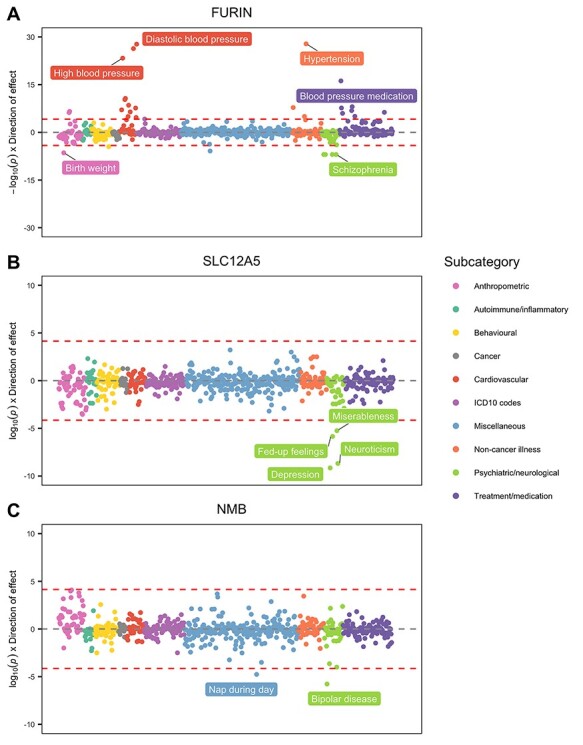
Bi-directional phenome-wide association plots depicting MR results between brain-derived gene expression and over 700 different traits and outcomes for (**A**) *FURIN*, (**B**) *SLC12A5* and (**C**) *NMB*. Points are coloured and clustered based on the subcategories of traits.

MR and genetic colocalization analyses were undertaken systematically on 700 outcomes using findings from large-scale GWAS consortia and the UK Biobank study (Supplementary Material, Table S12). Undertaking MR analyses at each of the 83 brain tissue genes and 700 complex traits from across the phenome identified 52 genes which had a genetically predicted effect on more than one outcome (Supplementary Material, Tables S13 and S14). There were established psychiatric loci which appear to be highly pleiotropic, such as *FURIN* (genetically predicted to influence 28 traits, Fig. 4A) and *MAPT* (predicted effects on 33 traits) based on the phenome-wide corrections of *P* < 7.14 × 10^−5^. These findings therefore suggest that they should be deprioritized as therapeutic targets for functional follow-up studies, as inhibiting their expression may result in unanticipated adverse effects.

In contrast, our analyses did not flag the potential adverse effects for various loci in this analysis. This included genes whose genetically predicted expression was associated with schizophrenia, such as *CLCN3* (*P* = 5.99 × 10^−7^) and *ABCB6* (*P* = 9.48 × 10^−7^) and *SLC12A5*, which was associated with depression (*P* = 7.30 × 10^−10^, Fig. 4B), serine protease 36 (*PRSS36*) which was associated with Alzheimer’s disease (*P* = 7.30 × 10^−8^) and neuromedin B (*NMB*) which was associated with bipolar disease (*P* = 1.70 × 10^−6^, Fig. 4C).

## Discussion

We have applied an integrative approach using brain-derived molecular datasets to highlight biologically relevant genes which may putatively influence risk of eight neurological and psychiatric outcomes. This analysis provided evidence suggesting that genetically predicted gene expression in brain tissue may influence at least one of these traits at 83 genetic loci. Integrating DNA methylation data obtained from brain samples provided further evidence that epigenetic factors and gene expression may play a role in trait variation at nine of these loci. Furthermore, we repeated our integrative framework using gene expression data derived from whole blood to examine whether these putative effects would have been detected using this proxy tissue type. In our study, a large proportion of brain-derived associations (~60%) survived a heuristic threshold of *P* < 0.05 in blood, corroborating the use of blood as a starting point for target identification. Conversely, despite the dramatically lower sample size for brain tissue (less than 30×), there were ~40% of the genetically predicted brain tissue effects that were not detected in blood based on this threshold. This may suggest that these effects depend strongly on using data from a tissue-type which is biologically relevant to the outcomes assessed. However, it is important to consider that despite brain being the primary tissue of interest in this study, other tissues may be more pertinent in some instances. For example, lung and spleen tissues have previously been shown to be enriched amongst Alzheimer’s disease loci (17). Finally, we investigated potential pleiotropic effects at these loci by assessing their association with 700 outcomes from large-scale GWAS and the UK Biobank study.

Tissue- and cell-dependent gene expressions have been previously shown to help elucidate the biological processes and functions of genes (22,23). There are various genes highlighted in our study which only provided evidence of a genetically predicted effect on outcomes when leveraging brain-derived gene expression. For instance, the genetically predicted effects between *KIFC2, NSF* and *TRIOBP* with intelligence appeared to depend on using expression data from the brain tissue, despite the roughly 30-fold increase in the sample size with whole blood. Furthermore, we identified other genes that provided much stronger evidence of association using brain tissue in comparison with whole blood. This included genes such as *SLC12A5*, which showed evidence of a genetically predicted effect on depression and neuroticism (*P* = 7.13 × 10^−10^ and *P* = 2.10 × 10^−9^ respectively), as well as *PCDHA8*, which provided evidence of genetically predicted effect on schizophrenia risk (*P* = 6.04 × 10^−6^). All these loci have been shown to have a role in neuronal maintenance and neurotransmitter release (see details in Table 1).

**Table 1:** Function and previous literature on genes mentioned in the discussion

Gene name	Putative biological function and previous transcriptome-wide studies	Current study
***CLCN3***	Studies (18,35) using expression data in adult brain tissue from the dorsolateral prefrontal cortex of the CMC (35) showed that genetic variants encoding *CLCN3* are upregulated by schizophrenia risk allele. *CLCN3* is a brain-expressed chloride ion channel, implicated in controlling fast excitatory glutamatergic transmission (65). *CLCN3^−/−^* mice show neurological symptoms such as blindness, motor coordination deficits and spontaneous hyperlocomotion as well progressive degeneration of the retina, hippocampus and ileal mucosa (66).	DNA methylation and expression of *CLCN3* associated with a higher risk of schizophrenia (β: 0.28, se = 0.06, *P* = 5.99 × 10^−7^ in brain tissue)
***FURIN*** (paired basic amino acid cleaving enzyme)	Previous eQTL studies have shown the expression of *FURIN* to be associated with the risk of schizophrenia (67,68). One of these studies (68) integrated genetic associations from schizophrenia GWAS and brain eQTL from 193 normal human subjects, using different approaches such as Sherlock, summary MR, DAPPLE, Prix Fixe and NetWAS. The other study (67) performed this analysis using the summary MR package, only using brain tissue samples from the CMC (*N* = 467). A study showed that altering expression of *FURIN* changed neurodevelopment in zebrafish, and knockdown of *FURIN* in human progenitor cells resulted in abnormal migration (35). *FURIN* processes precursor proteins to mature forms, including brain-derived neurotrophic factor (*BDNF*), whose downregulation has been associated with a higher risk of schizophrenia (69).	Expression of *FURIN* associated with risk of schizophrenia (β: −0.25, se = 0.05, *P* = 1.05 × 10^−7^ in brain tissue)
***KIFC2***	*KIFC2* was implicated in genetically predicted intelligence through eQTL mapping in two different studies (17,70). The *KIFC* gene encodes C-terminal kinesin-related motor protein that has been found to be specifically expressed in adult neurons in mice and has been shown to be involved in microtubule-dependent retrograde axonal transport in dendrites (71).	Expression of *KIFC2* associated with intelligence (β: −0.05, se = 0.01, *P* = 9.93 × 10^−8^ in brain tissue)
***NMB***	*NMB* encodes a family of bombesin-like peptides which are mainly expressed in the hypothalamus, stomach and colon (72). A study using summary MR integrating their GWAS summary statistics (73) findings with eQTL data from the dorsolateral prefrontal cortex (35) and whole blood (6), showed that genetic variants encoding *NMB* are upregulated by the bipolar risk allele.	Expression of *NMB* associated with bipolar disease (β: 0.30, se = 0.06, *P* = 1.70 × 10^−6^ in brain tissue)
***NSF***	The most recent genome-wide meta-analysis for intelligence (17) implicated *NSF* in predicting intelligence using methods such as SNP-based genome-wide association test, gene-based genome-wide association test (using aggregate effect of all SNPs in a gene) and eQTL mapping. The *NSF* gene encodes ATPase N-ethylmaleimide-sensitive factor, which plays an important role in vesicle fusion events and in regulating neurotransmitter release kinetics (74).	Expression of *NSF* associated with intelligence (β: 0.05, se = 0.01, *P* = 1.70 × 10^−8^ in brain tissue)
***PCDHA8***	A study (16) which applied multiple trait colocalization to identify regulatory effects at GWAS risk loci for schizophrenia identified a shared causal variant affecting the risk of schizophrenia through DNA methylation and expression of *PCDHA8*. This study used methylation data from postmortem tissue of the dorsolateral prefrontal cortex of non-psychiatric control donors (*N* = 121), gene expression data from dorsolateral prefrontal cortex of patients in a case-control study collected by the CMC sample (48) and the schizophrenia GWAS by Ripke *et al*. (75). *PCDHA8* was associated with schizophrenia risk which is a member of the protocadherin family of genes that are involved in the establishment and maintenance of neuronal connections in the brain (76).	Expression of *PCDHA8* associated with the risk of schizophrenia (β: 0.11, se = 0.02, *P* = 6.04 × 10^−6^ in brain tissue)
***PRSS36*** (serine protease 36)	*PRSS36* has been implicated via eQTL association in the hippocampus, which is highly affected early in Alzheimer’s disease. Furthermore, a summary-based tissue-specific transcriptome-wide association study (77) using a factor polygenic QTL analysis, jointly modelling gene expression across tissues and individuals, found brain-specific expression of *PRSS36* to be associated with Alzheimer’s disease.	Expression of *PRSS36* associated with risk of Alzheimer’s disease (β: −0.02, se = 0.003, *P* = 7.30 × 10^−8^ in brain tissue)
***SLC12A5***	A previous integrative analysis using a different brain eQTL and mQTL dataset found that the expression of the *SLC12A5* colocalized the risk of neuroticism (78). Furthermore, it has been reported to increase the risk of other brain-related disorders, such as epilepsy (79). Changes in *SLC12A5* expression were not identified as conferring risk to MDD in a Sherlock analysis (80), integrating the genetic associations from a MDD GWAS (42) and brain eQTL dataset (46). The *SLC12A5* gene encodes a neuron-specific transmembrane cotransporter (81), which plays a role in mediating fast synaptic inhibition.	Depression (β: 0.03, se = 0.004, *P* = 7.13 × 10^−10^). Neuroticism (β: 0.04, se = 0.007, *P* = 2.10 × 10^−9^ in brain tissue)
***SLC25A12***	None of the integrative analyses (78,82) have identified methylation or expression of *SLC25A12* to affect risk of neuroticism. *SLC25A12* encodes a calcium-binding solute carrier in the inner mitochondrial membrane that is essential for energy homeostasis (26).	DNA methylation and expression of *SLC25A12* associated with neuroticism score (β: 0.06, se = 0.01, *P* = 6.42 × 10^−6^ in brain tissue)
***TRIOBP-1***	A GWAS study (17) which performed functional annotation of top hits for intelligence identified a SNP nearest to the *TRIOBP* gene. Furthermore, *TRIOBP* contained three exonic non-synonymous variants associated with intelligence. *TRIOBP* has also been identified in gene-based association analyses of brain volumetric phenotypes (83). *TRIOBP* encodes proteins with a role in neural tissue development and controlling cytoskeleton organization, cell motility and cell growth (84).	Expression associated with intelligence (β: 0.03, se = 0.01, *P* = 3.59 × 10^−8^ in brain tissue)

Abbreviations: MDD, major depressive disorder; qTL, quantitative trait locus.

We also found evidence suggesting that both gene expression and DNA methylation may be involved along the causal pathway between the genetic variant and trait variation at nine of the 83 associated loci. These findings build upon previous research that has harnessed QTL datasets from the brain tissue (24,25), suggesting a coordinated system of effects between the regulatory elements (e.g. transcription factors) and gene transcription, which is consistent with causality. These loci included *SLC25A12,* where a PPA of 0.96 suggested that genetic variation here influences proximal DNA methylation, *SLC25A12* expression and neuroticism risk. This gene encodes a calcium-binding solute carrier in the inner mitochondrial membrane, which is essential for energy homeostasis (26). Polymorphisms in the *SLC25A12* gene have been shown to strongly associate with autism in candidate gene studies (27–30). Furthermore, its expression has been associated with neurite outgrowth and is upregulated in the prefrontal cortex of autistic individuals (31). Although we did not study autism, neuroticism and autism are genetically (32) and phenotypically correlated (33,34).

Phenome-wide investigations of the genes identified using brain tissue provide indications of possible pleiotropic effects, which may suggest that therapeutically targetting them could be detrimental to other health outcomes. For example, *FURIN*, whose expression was linked with schizophrenia risk (*P* = 1.05 × 10^−7^), was also associated with traits such as high blood pressure and diastolic blood pressure but with the opposite direction of effect. In contrast, we did not find evidence of potential adverse effects for potentially novel targets, such as *SLC12A5* (depression, *P* = 7.13 × 10^−10^), *NMB* (bipolar disorder, *P* = 1.70 × 10^−6^) and *PRSS36* (Alzheimer’s disease, *P* = 7.30 × 10^−8^), deeming them potentially promising and worthwhile druggable loci. Moreover, we identified evidence to support previously implicated targets, such as *CLCN3* (schizophrenia, *P* = 5.99 × 10^−7^), which have been reported to play a role in the control of glutamatergic transmission (35). As with all analyses in this study, however, these genes are merely examples to highlight potentially translatable opportunities from our findings (more examples are provided in Supplementary Material).

A previous study by Qi *et al*. (36) reported that the genetic effects at the top *cis-*eQTLs and mQTLs are highly correlated between independent brain and blood samples (*r*^2^ = 0.7 and *r*^2^ = 0.78, respectively), supporting the use of whole blood as a proxy for gene discovery efforts in brain-related traits. We integrated whole blood-derived expression data from eQTLGen with the GWAS summary statistics of examined traits to prioritize potential candidates which we may have been underpowered to detect in brain tissue. This analysis identified 37 candidate loci for future follow-up once larger samples of brain tissue are available. Whilst these findings support the utility of whole blood as proxy for more pertinent tissue-types, our analyses also uncovered evidence suggesting that various genetically predicted effects may still be overlooked by not using brain tissue data (~40%). This therefore supports future endeavours aiming to derive molecular datasets using brain tissue in larger sample sizes. Subsequently, this will reduce false positives rates to which smaller sample sizes may be prone to as well as validate findings such as those in our study. That said, whole blood may still be a useful proxy when sufficient samples of brain tissue data are not accessible. Genes identified in this study such as *SLC12A5* and *NSF* are such examples where whole blood appeared to be a valid proxy in our study, given that findings from GTEx suggest that they are expressed much more in brain tissue compared with whole blood (Supplementary Material, Figs S1 and S2).

In terms of future work, we performed our analysis instrumenting gene expression with *cis*-variants as *trans*-effects may be more prone to horizontal pleiotropy. The small variance explained by individual *trans-*eQTL/mQTL necessitates larger sample sizes for their identification and use in MR analyses (37). Furthermore, the lack of full summary data for the mQTL data poses a limitation for the multiple trait colocalization analysis. Our findings therefore prioritize these loci for future evaluations by epigenetic studies to determine whether a disruption to transcription binding sites or chromatin compaction has downstream implications for neurological trait variation. For ADHD, we may have lacked the power to identify many disease-associated loci due to the small sample of the corresponding GWAS. Due to modest sample sizes for current molecular datasets, largely attributed to the cost of sequencing arrays, we are unable to robustly delineate the causal sequence of events between molecular traits and outcomes. For example, it is plausible in our study that the outcome may have an effect on DNA methylation and/or gene regulation rather than the converse direction of effect as evaluated in our framework.

Furthermore, although the integration of various biological datasets in many situations can help prioritize genes responsible for disease risk, it is difficult to isolate the causal gene(s) due to the mechanistic co-regulation of genes, where the expression of two or more genes is correlated due to their regulation by the same eQTL (i.e. mechanistic co-regulation). Moreover, co-regulation may imply that the genes are involved in the same pathway, making it plausible that multiple genes play a causal role. Although genetic colocalization analyses can mitigate the likelihood of false positives due to linkage structure at the loci, functional studies are required to definitively rule out horizontal pleiotropy as a potential explanation for our results. Conducting phenome-wide association studies in other tissues may be useful in identifying pleiotropic signals not confined to brain tissue. Likewise, applying our framework using tissue-specific protein QTL should prove useful in terms of identifying evidence potentially overlooked by eQTL (38). Lastly, there are limitations in using MR, such as the presence of non-transmitted genetic effects [3, 4] known to violate the independence assumption of MR [5]. Future developments in family studies may aid in disentangling transmitted environmental effects from those of genetic origin.

Our findings emphasize the importance of leveraging molecular datasets derived from biologically relevant tissues to develop insight into the causal pathway for trait-associated variants. Our results provide a prioritized list of candidate genes which future studies can investigate to better characterize the biological mechanisms which influence the neurological traits and risk of psychiatric disorders.

## Materials and Methods

### Data sources

#### GWAS summary data

We obtained the beta coefficient (log odds ratio), standard error, effect allele, alternate allele, effect allele frequency and sample size from the summary statistics of eight independent GWAS for the following brain-related diseases/traits: Alzheimer’s disease (*N*_cases_ = 71 880), ADHD (*N*_cases_ = 20 183) (39), bipolar disorder (*N*_cases_ = 20 129) (40), depression (*N*_cases_ = 170 756) (41,42), insomnia (*N*_cases_ = 208 716) (43), intelligence (*N* = 279 930) (17), neuroticism (score) (*N* = 374 317) (44) and schizophrenia (*N*_cases_ = 33 426) (40). As the GWAS summary statistics for intelligence by Savage *et al*. (17) have been conditioned on socioeconomic status (SES), we also include the effect estimates of identified genes from a GWAS of fluid intelligence score without adjustment for SES in Supplementary Material. Details on GWAS datasets are described in Supplementary Material, Table S1.

#### Brain tissue-derived quantitative trait loci data

We obtained eQTL summary data from a meta-analysis (*n*_eff_ = 1194) of GTEx brain (*n* = ~233) (45), Common Mind Consortium (CMC) (*n* = 467) (35) and Religious Orders Study and Memory Aging Project (ROSMAP) (*n* = 494) (46) undertaken by Qi *et al*. Only SNPs within 1 Mb distance from each probe (*n* = 28 538) were available (*cis*-QTLs). Additionally, from Qi *et al*. (36), we extracted mQTL summary statistics from a meta-analysis of three brain-derived datasets: brain cortical region from ROSMAP study (*n*_ind_ = 468, *n*_probe_ = 420 103, *n*_snp_ = 5 million) (36); fetal brain (*n*_ind_ = 166, *n*_probe_ = 26 840, *n*_snp_ = 0.3 million) (47) and frontal cortex region (*n*_ind_ = 526, *n*_probe_ = 138 917, *n*_snp_ = 1.5 million) (48) (Supplementary Material, Table S2). DNAm levels in all these studies were based on the Illumina Human Methylation 450 K array. In ROSMAP, only SNPs within 5 kb of each DNA methylation probe were available. In the Hannon *et al*. data, only SNPs within 500 kb distance from each probe and with P < 1.0 × 10^−10^ were available. In the Jaffe *et al*. data, only SNPs within 20 Kb distance from each probe and FDR < 0.1 were available. In the CMC sample, 209 participants had a diagnosis of schizophrenia, whilst 288 in ROSMAP had a diagnosis of Alzheimer’s disease.

#### Whole blood-derived eQTL data

We obtained eQTL data from a large-scale meta-analysis (6) in non-transformed peripheral blood samples (*N* = 31 684) from 37 cohorts (Supplementary Material, Table S2). Data from the eQTLGen consortium include association summary statistics for 10 023 016 SNPs and 19 251 genes (resource accessed on 18 October 2018). *Cis*-eQTLs were defined based on a distance less than 1 Mb from their associated genes probe. Several of these cohorts included participants with cardiovascular disease or/and psychiatric disorders (Supplementary Material, Table S2). Further details are available at https://www.eqtlgen.org/.

### Statistical analysis

#### Brain-derived gene expression analysis

Linkage disequilibrium clumping was undertaken with PLINK using *r*^2^ < 0.01 and based on a reference panel of European individuals from the 1000 genomes phase 3 project (49). When we were only able to instrument a gene’s expression using a single independent eQTL, the Wald ratio method was used to estimate the MR effects (50). When two or more independent eQTL were available for MR analyses, we used the inverse variance weighted (IVW) method (51). Single SNP MR analyses may be prone to higher false discovery rates due to the linkage disequilibrium between the causal eQTL at a locus and a separate but correlated variant responsible for changes in trait variation. As such, results which survived multiple testing comparisons based on single SNP analyses were evaluated using genetic colocalization to discern whether the effects were attributed to the same underlying variant. This was assessed using the ‘coloc’ R package (52) where a PPA > 0.8 was used as the evidence of colocalization. Effects which survived multiple testing (Bonferroni-corrected threshold of *P* < 0.05/number of tests) were carried through to subsequent downstream analyses. We also compared the genes identified in our study for schizophrenia to those identified in two transcriptome-wide association studies (3,18) (Supplementary Material, Table S6).

#### Detecting shared aetiology between traits and brain region enrichment analysis

To examine potential shared aetiology between the eight traits, we investigated how the genetically predicted effects of identified genes for these traits clustered based on Euclidean distance matrix computation using the R package ‘dist’ (53). A heatmap to visualize these clusters was generated using the package ‘pheatmap’ (54). Enrichment analyses for clusters of genes associated with the same trait were evaluated using the MAGMA approach (55), which was undertaken as part of the FUMA platform (56). Genes of interest were tested against 19 283 protein-coding gene sets obtained from MsigDB (i.e. hallmark gene, sets, positional gene sets, curated gene sets, motif gene sets, computational gene sets, GO gene sets, oncogenic signatures and immunologic signatures) and WikiPathways, using hypergeometric tests (56). Using data from GTEx v7 (45), we assessed whether there was evidence of gene expression enrichment clustering in specific regions of the brain.

#### A comparison between brain-derived gene expression effects and those identified using whole blood

We repeated our analysis pipeline using eQTL derived from eQTLGen in whole blood to compare the effect estimates between brain and blood-derived analyses. This allowed to highlight effects which were much stronger in brain-derived tissue, particularly given the discrepancy in sample sizes (*n* = 1194 in brain and *n* = 31 684 in blood). A threshold of *P* < 0.05 was used as a heuristic in this analysis to highlight associations with a strong evidence of differential expression in either brain or blood.

#### Multiple trait colocalization analysis using brain-derived DNA methylation effects

We used multiple trait colocalization to discern whether there was evidence that DNA methylation, gene expression and cognitive or psychological trait of interest are all influenced by the same causal variant at each loci investigated. A PPA > 0.8 was used as evidence of genetic colocalization, this time using the ‘moloc’ R package (16). Analyses were run multiple times to investigate the DNA methylation at CpG sites within 100 kb distance of gene coordinates. For loci where there were two or more independent eQTLs based on linkage disequilibrium clumping, we evaluated each signal in turn by conditioning out separate effects using GCTA-COJO (57).

#### Phenome-wide analysis

We undertook hypothesis-free analyses of genes identified in our primary analysis using brain eQTL data to elucidate pleiotropic effects. A Bonferroni-corrected threshold of *P* < 7.14 × 10^−5^ (i.e. 0.05/700 traits) was used as a heuristic to highlight putative pleiotropic effects for target genes, provided they were druggable based on previous evidence as described in the study by Leyden *et al*. (58). Briefly, this comprised of a curated list obtained from four data-driven drug discovery endeavours, which evaluated whether the product of protein-coding genes could be altered using targeted therapeutics (59–62). These findings were derived to flag the possible adverse side effects as well as the multi-purposing potential of therapeutic interventions of these genes.

## Supplementary Material

Supplementary_Table_ddab016

Copy_of_Supplementary_Tables_Final_TGR_(3)_ddab016

## Data Availability

All GWAS datasets on primary outcomes analyzed in this study are available publicly as described in Supplementary Material, Table S1. Analyses were conducted using R (version 3.3.1) unless stated otherwise. MR analyses were undertaken using the ‘TwoSampleMR’ package (63) where all the outcomes analyzed in our phenome-wide association analyses are available. Plots were generated using the ‘ggplot2’ package (64).
